# Successful Treatment of Angiolymphoid Hyperplasia with Eosinophilia Associated with Scalp Demodicosis Using Cryotherapy and Topical Metronidazole

**DOI:** 10.18295/squmj.3.2024.020

**Published:** 2024-08-29

**Authors:** Salma T. Al Kharusi, Aya A. Al Lamki, Raqiya M. Al Rajaibi, Zakiya I. Al Ajmi

**Affiliations:** 1 Dermatology Department, Rustaq Polyclinic, Rustaq, Oman; 2 Department of Histopathology, Royal Hospital, Muscat, Oman

**Keywords:** Angiolymphoid Hyperplasia with Eosinophilia, Mite, Scalp, Kimura Disease, Cryosurgery, Metronidazole, Case Report, Oman

## Abstract

Angiolymphoid hyperplasia with eosinophilia (ALHE) is a rare, benign, vasoproliferative tumour. We report a 25-year-old female patient who reported in 2021 to a dermatology clinic in Rustaq, Oman, with multiple, grouped, erythematous dome-shaped papules and nodules of 6 months duration on the left temporo-occipital region. Biopsy findings were consistent with a diagnosis of ALHE with evidence of Demodex mite infestation in the sebaceous ducts. The patient demonstrated significant improvement following 7 weeks of treatment with multiple cryotherapy sessions and topical application of metronidazole gel. This case suggests that scalp demodicosis may represent a novel trigger for the development of ALHE.

Angiolymphoid hyperplasia with eosinophilia (ALHE) is a benign, uncommon, proliferation of blood vessels of uncertain aetiology and pathogenesis.^1^–^3^ It is characterised by the presence of single or multiple, cutaneous, or subcutaneous, red-to-brown coloured papules or nodules commonly located in the head and neck region. Although many treatment modalities have been suggested, no standardised approach has yet been established.^2^ We describe a case of ALHE alongside scalp demodicosis who was successfully treated.

## Case Report

**Figure 1:**
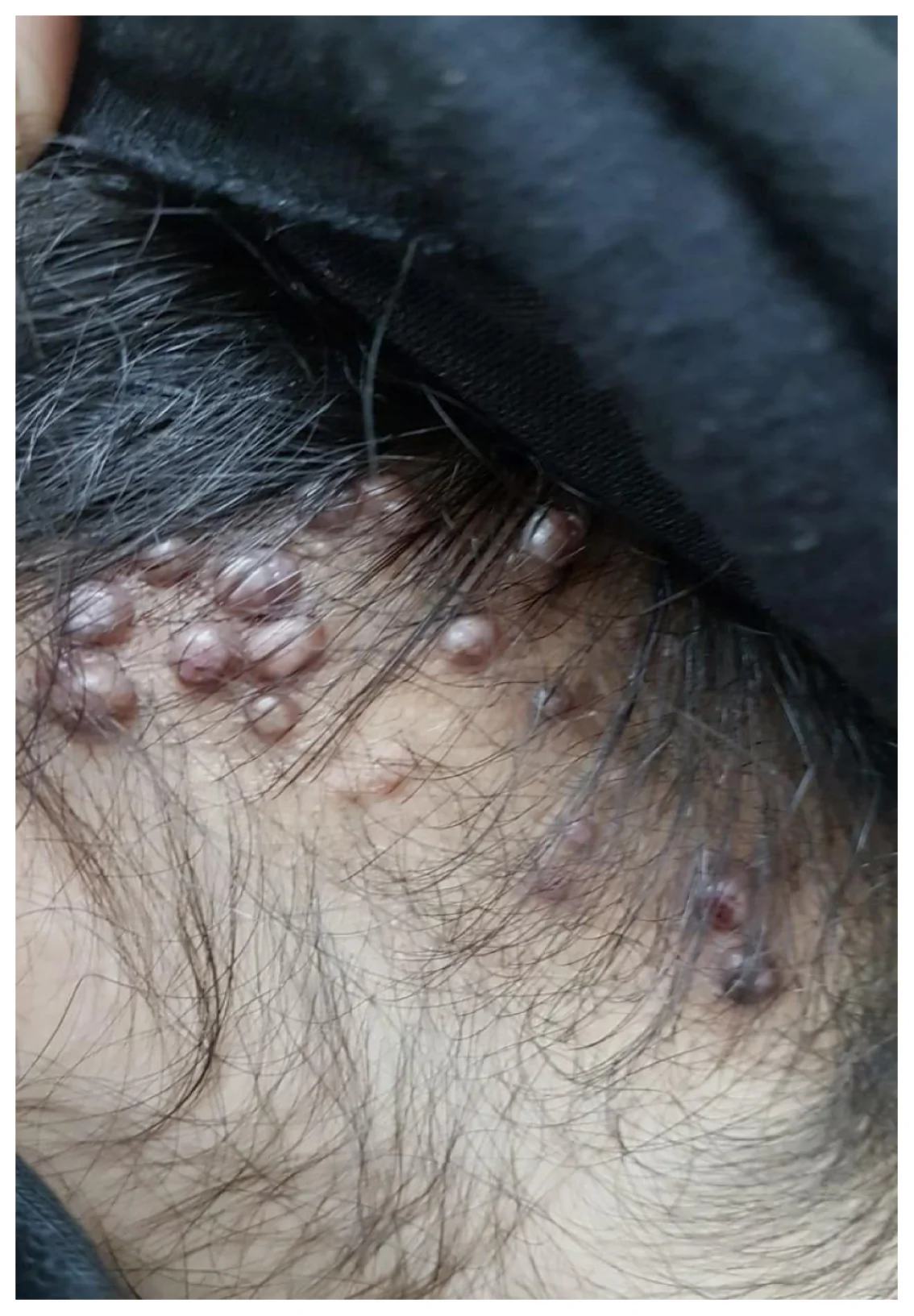
Photograph of the left temporo-occipital region showing multiple erythematous papules and nodules (angiolymphoid hyperplasia with eosinophilia lesions before treatment).

A 25-year-old female patient presented to a dermatology clinic in Rustaq, Oman, in 2021 with multiple, itchy, pearly papules on her scalp of approximately 6 months duration. She reported ulcerations and discrete bleeding after scratching due to pruritus and denied any history of systemic symptoms or local trauma. Clinical examination of the scalp showed multiple, grouped, erythematous papules and nodules, with an average diameter of 1 cm, located on the left temporo-occipital region [Figure 1]. The systemic examination was unremarkable and there was no evidence of regional or systemic lymphadenopathy. A complete blood count (including eosinophils), renal function test, serum immunoglobulin E levels, HIV screening and urine analysis were all normal. The patient had not received any previous treatments for these lesions prior to presentation to the current clinic.

**Figure 2:**
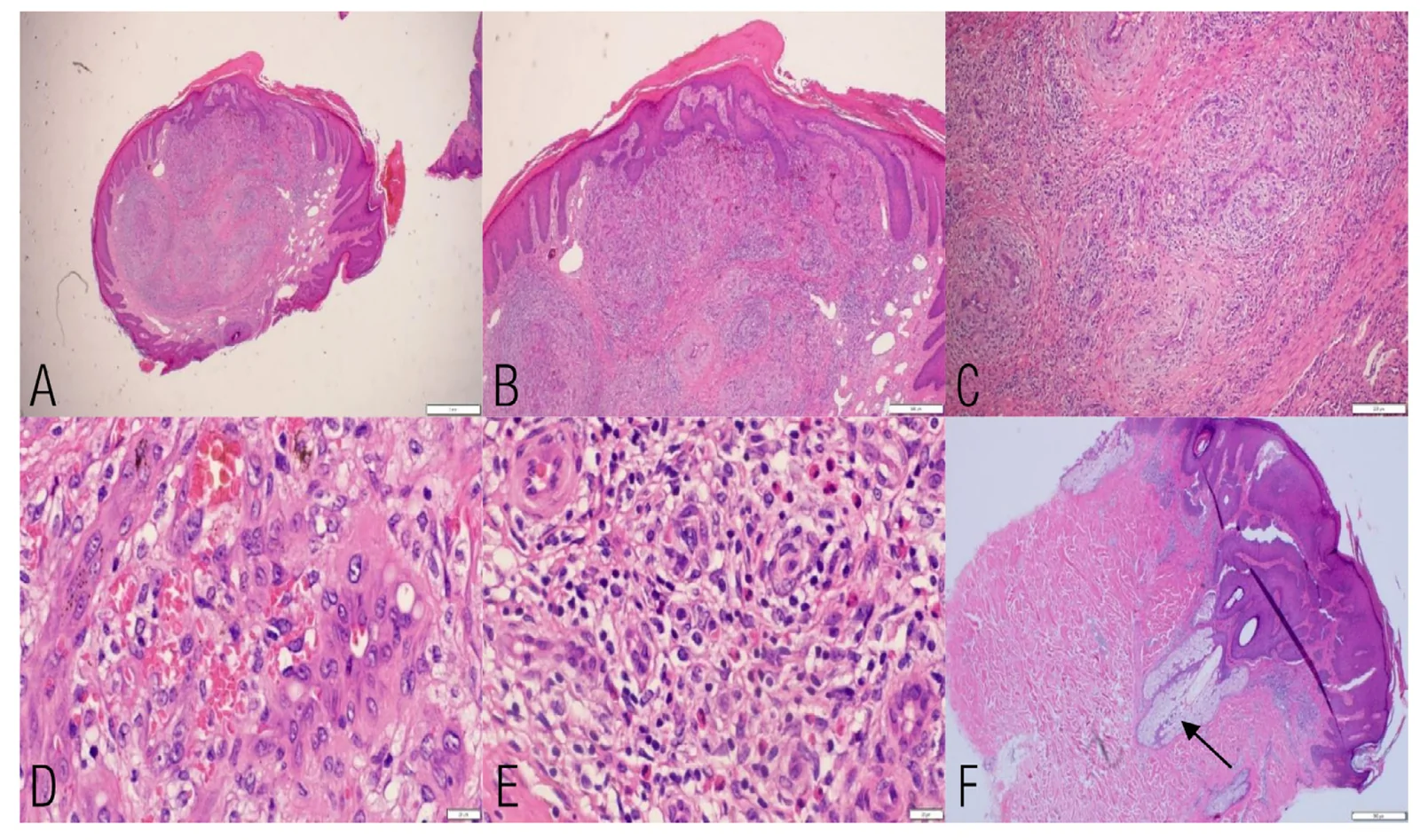
Haematoxylin and eosin stains of the biopsied lesion at (A) × 2 magnification showing dense deep dermal and subcutaneous proliferation; (B & C) × 5 and × 10 magnification, respectively, showing the lobular proliferation is composed of variable-size blood vessels surrounded by scattered inflammatory cells; (D) × 40 magnification showing the lining endothelial cells exhibiting enlarged vesicular nuclei and vacuolated cytoplasm with evidence of haemorrhage; (E) × 40 magnification showing the surrounding inflammation is composed of lymphocytes admixed with many eosinophils and (F) × 4 magnification showing Demodex mites (arrow) in the sebaceous ducts.

Following a biopsy of one of the lesions, the histopathological examination revealed the proliferation of variable-sized blood vessels lined by plump histiocytoid endothelial cells, as well as inflammatory infiltrates comprising lymphocytes and eosinophils in the dermis [Figure 2A–E]. The biopsy also revealed evidence of Demodex mite infestation in the sebaceous ducts [Figure 2F]. Based on these histopathological and clinical features, a diagnosis of ALHE and scalp demodicosis was made.

**Figure 3:**
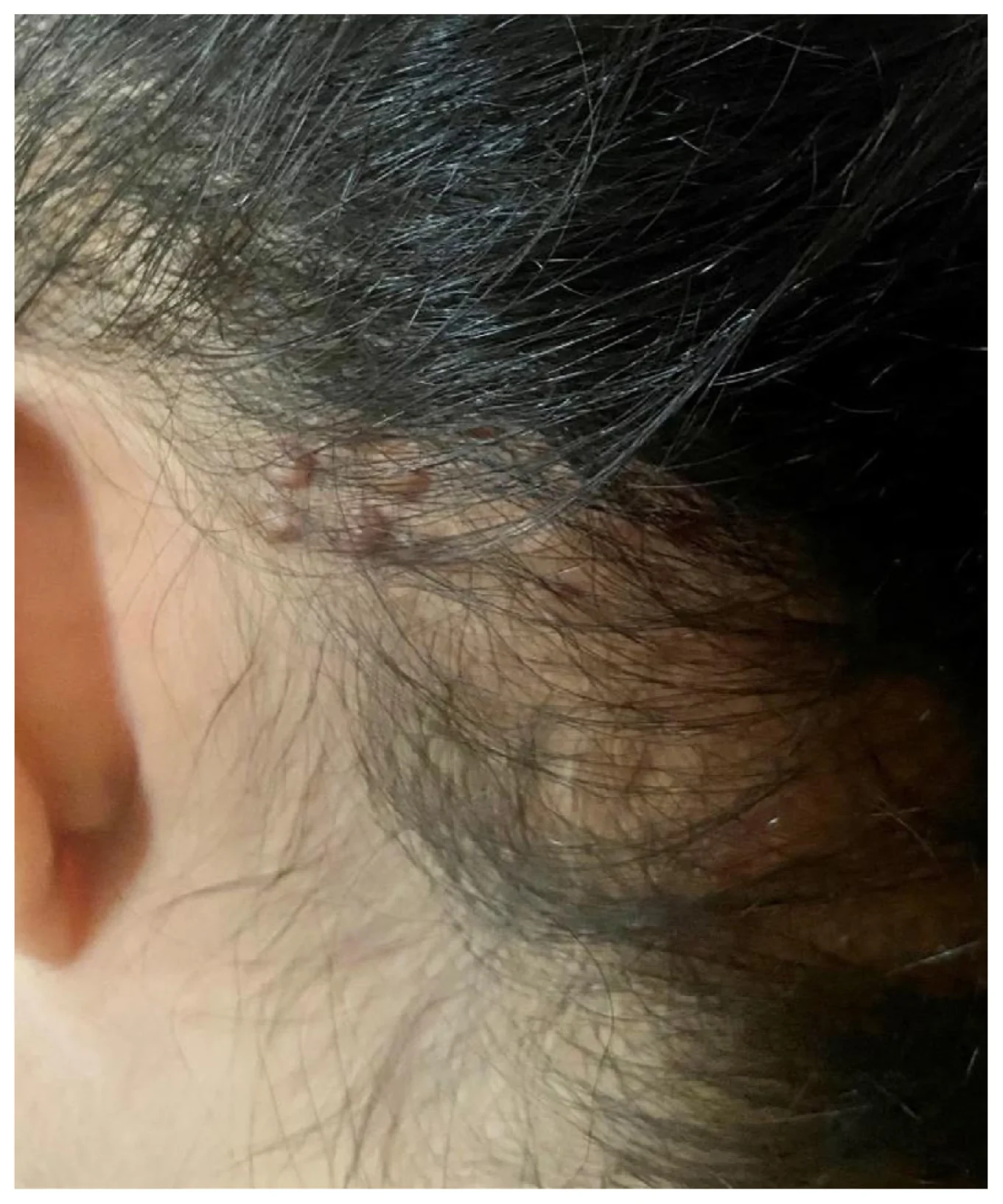
Photograph of the left temporo-occipital region showing significant resolution of angiolymphoid hyperplasia with eosinophilia lesions following 7 weeks of treatment using cryotherapy and topical metronidazole.

The patient was treated with 10 sessions of cryotherapy (each session consisted of 2 freeze-thaw cycles per week for each lesion). In addition, twice-daily application of a topical metronidazole gel was incorporated into the treatment regimen. The patient showed remarkable clinical improvement within 7 weeks [Figure 3]. She was subsequently followed-up for the next year with no signs of recurrence or new lesions appearing.

Informed patient consent regarding the publication of this case was obtained.

## Discussion

Also known as epithelioid haemangioma, ALHE is a rare, benign, vasoproliferative neoplasm first described in 1969. It is common in the Asian population and usually affects middle-aged adults.^1^–^3^

ALHE usually presents as single or multiple well-defined, erythematous or brownish papulonodular lesions and can be pulsatile.^4^ The condition is usually localised to the head and neck, mainly in the periauricular region; however, it has rarely been reported to affect other parts of the body, such as the colon, hands, penis and oral mucosa.^5^^,^^6^ Overall, ALHE can be asymptomatic but may also present with spontaneous bleeding, itchiness or pain.^2^

Currently, the aetiology and pathogenesis of ALHE are not fully understood. The commonly accepted hypothesis is that it is a reactive vascular hyperplasia to certain stimuli, such as trauma, hyperoestrogenism, vascular malformation, reaction to insect bite and infections such as scabies or HIV.^7^–^9^ However, several researchers have raised concerns with this explanation due to the presence of clonal T-cell populations in many cases, with some authors proposing that certain types of ALHE might be due to a benign- to low-grade malignant T-cell lymphoproliferative disorder.^10^^,^^11^

The differential diagnoses of ALHE include epithelioid haemangioendotheliomas, pyogenic granulomas, Kaposi sarcomas and Kimura disease (KD).^4^ The latter is considered the main differential diagnosis of ALHE due to their clinical and histopathological similarities.^12^ Previously, ALHE and KD were assumed to be the same disorder, but now these two entities can be distinguished due to the distinctive features of the latter condition, as KD presents with subcutaneous masses in the head and neck region, alongside regional and, rarely, systemic lymphadenopathy, peripheral eosinophilia and elevated serum immunoglobulin E levels, and is infrequently associated with nephrotic syndrome.^12^–^15^ Although ALHE and KD are two separate diseases, there are some reported cases of overlapped presentation, suggesting that both diseases could be a variant of the same reactive vascular lymphoid proliferation disorder.^16^

The histopathologic picture of the lesion demonstrates deep dermal and subcutaneous lobular proliferation of capillary size blood vessels of variable sizes. These are lined by plump epithelioid endothelial cells exhibiting enlarged vesicular nuclei and some with vacuolated cytoplasm. The surrounding stroma shows foci of haemorrhage and moderate infiltration by lymphocytes and eosinophils. There are no lymphoid follicles identified (KD demonstrates a marked lymphoid follicular hyperplasia). The inflammatory cells may penetrate the lumen of blood vessels, blocking or rupturing them.^12^ This phenomenon is not seen in the current case’s biopsy, though there is evidence of haemorrhage which may suggest vascular destruction elsewhere in the lesion.

Spontaneous regression of ALHE is sometimes reported.^5^ In other cases, choice of treatment depends on the position, size, depth and number of lesions, in addition to histological features and skin pigmentation. Many potential treatment modalities have been suggested, with variable success; however, recurrence is commonly noted. Thus, surgical excision remains the best modality of treatment due to low recurrence rates.^2^ Other modalities include administration of topical and intralesional corticosteroids, topical tacrolimus or imiquimod, oral isotretinoin, interferon alpha-2b, radiotherapy, thalidomide, photodynamic therapy, propranolol, laser therapy (using neodymium-doped yttrium aluminium garnet, carbon dioxide, ultralong pulsed dye or copper vapor lasers), electrosurgery and cryosurgery.^4^^,^^15^–^23^

In the current case, given that superficial vascular proliferation was a major feature, cryotherapy was deemed the best treatment option as it causes necrosis of vascular lesions, provoking an inflammatory response and lesion clearance.^24^ In particular, cryotherapy is indicated for multiple ALHE lesions with a prominent vascular component or for lesions located in sites difficult for excision. One of the benefits of cryosurgery is the satisfactory cosmetic result with minimal scarring, as freezing allows for the collagen fibre network of the skin to remain intact.^25^

To the best of the authors’ knowledge, the current case represents the first report of ALHE associated with scalp demodicosis. Demodex mites have been implicated in other pathological conditions of the scalp, including dermatitis, sebaceous cysts, rosacea, carcinomas and seborrheic keratosis.^26^ Moreover, the interaction between the pilosebaceous unit cells and Demodex mite antigens is believed to affect the secretion of inflammatory cytokines, such as tumour necrosis factor-alpha and interleukin-8, and toll-like receptor expression.^27^ Such inflammatory triggers are critical for eosinophil recruitment, itself crucial in the development of ALHE. Eosinophil cytotoxic proteins, such as eosinophil cationic protein, are believed to play a role in AHLE angiogenesis.^9^

## Conclusion

ALHE represents a challenging clinical and histological diagnosis. Despite its benign nature, there is no established therapeutic modality for ALHE because of its uncertain aetiopathogenesis. In the current case, the treatment combination of cryotherapy and topical metronidazole gel was successful in resolving both the ALHE lesions as well as the Demodex mite infestation, presumed to be the primary trigger. The case presented herein serves to emphasise that cryotherapy can be considered a safe, effective and reliable treatment option for ALHE patients in which there is a prominent vascular component. Moreover, the potential role of Demodex mites in the pathogenesis of this condition should be considered in further research.
